# Author Correction: Generation of a CRISPR activation mouse that enables modelling of aggressive lymphoma and interrogation of venetoclax resistance

**DOI:** 10.1038/s41467-022-32817-9

**Published:** 2022-08-25

**Authors:** Yexuan Deng, Sarah T. Diepstraten, Margaret A. Potts, Göknur Giner, Stephanie Trezise, Ashley P. Ng, Gerry Healey, Serena R. Kane, Amali Cooray, Kira Behrens, Amy Heidersbach, Andrew J. Kueh, Martin Pal, Stephen Wilcox, Lin Tai, Warren S. Alexander, Jane E. Visvader, Stephen L. Nutt, Andreas Strasser, Benjamin Haley, Quan Zhao, Gemma L. Kelly, Marco J. Herold

**Affiliations:** 1 The State Key Laboratory of Pharmaceutical Biotechnology, Department of Hepatobiliary Surgery, The Affiliated Drum Tower Hospital of Nanjing University Medical School, School of Life Sciences, Nanjing University, Nanjing, Jiangsu China; 2 The Walter and Eliza Hall Institute of Medical Research, Melbourne, VIC Australia; 3 Department of Medical Biology, University of Melbourne, Melbourne, VIC Australia; 4 Department of Molecular Biology, Genentech, Inc., South San Francisco, CA USA; 5 School of Dentistry and Medical Sciences, Charles Sturt University, Wagga Wagga, NSW Australia

**Keywords:** Genetic engineering, Cancer, Lymphoma

Correction to: *Nature Communications* 10.1038/s41467-022-32485-9, published online 12 August 2022

In this article the affiliation details for Amy Heidersbach were incorrectly given as ‘School of Dentistry and Medical Sciences, Charles Sturt University, Wagga Wagga, NSW, Australia’ but should have been ‘Department of Molecular Biology, Genentech, Inc., South San Francisco, CA, USA’. The original article has been corrected.

