# Supplementary material for: Generation of a CRISPR activation mouse that enables modelling of aggressive lymphoma and interrogation of venetoclax resistance
Source: Nat Commun. 2022 Aug 12;13:4739. doi: 10.1038/s41467-022-32485-9 (PMC9374748; doi:10.1038/s41467-022-32485-9)
Supplement: Supplementary file 1 — Supplementary Information [file 41467_2022_32485_MOESM1_ESM.pdf]

Supplementary information

Generation of a CRISPR activation mouse that enables modelling of aggressive lymphoma and interrogation of venetoclax resistance.

Supplementary Fig. 1

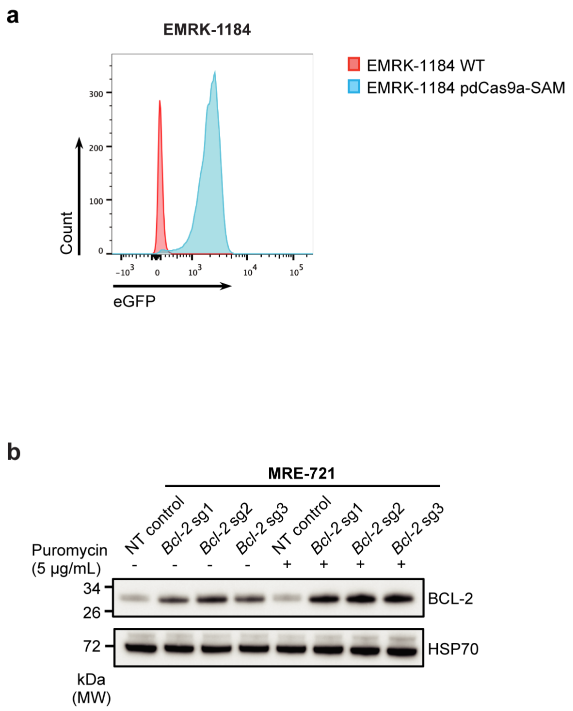

Supplementary Fig. 1 eGFP expression in the EMRK-1184 *Eµ-Myc* lymphoma cell line transduced with dCas9a-SAM construct. a eGFP expression in EMRK-1184 *Eµ-Myc* lymphoma-derived cell line was determined by flow cytometry. Cells were transduced with pdCas9A-SAM and sorted for eGFP positivity. Cells were then cultured for enrichment of eGFP positive populations and eGFP levels were determined by flow cytometry compared to uninfected EMRK-1184 cell line. **b** Western blot analysis for BCL-2 levels in manipulated *Eµ-Myc* lymphoma cell lines. MRE-721 *Eµ-Myc* lymphoma-derived cell lines were transduced with pdCas9a-SAM plus non-targeting control (NT) sgRNAs or pdCas9a-SAM plus *Bcl-2* sgRNAs. Cell lysates were harvested from transduced cell lines, before or after puromycin-selection and expression of the indicated proteins was examined by Western blotting to confirm that puromycin treatment does not impact BCL-2 expression levels. Probing for HSP70 served as a loading control. 2 independent experiments were repeated with similar results. Source data are provided as a Source Data file.

Supplementary Fig. 2

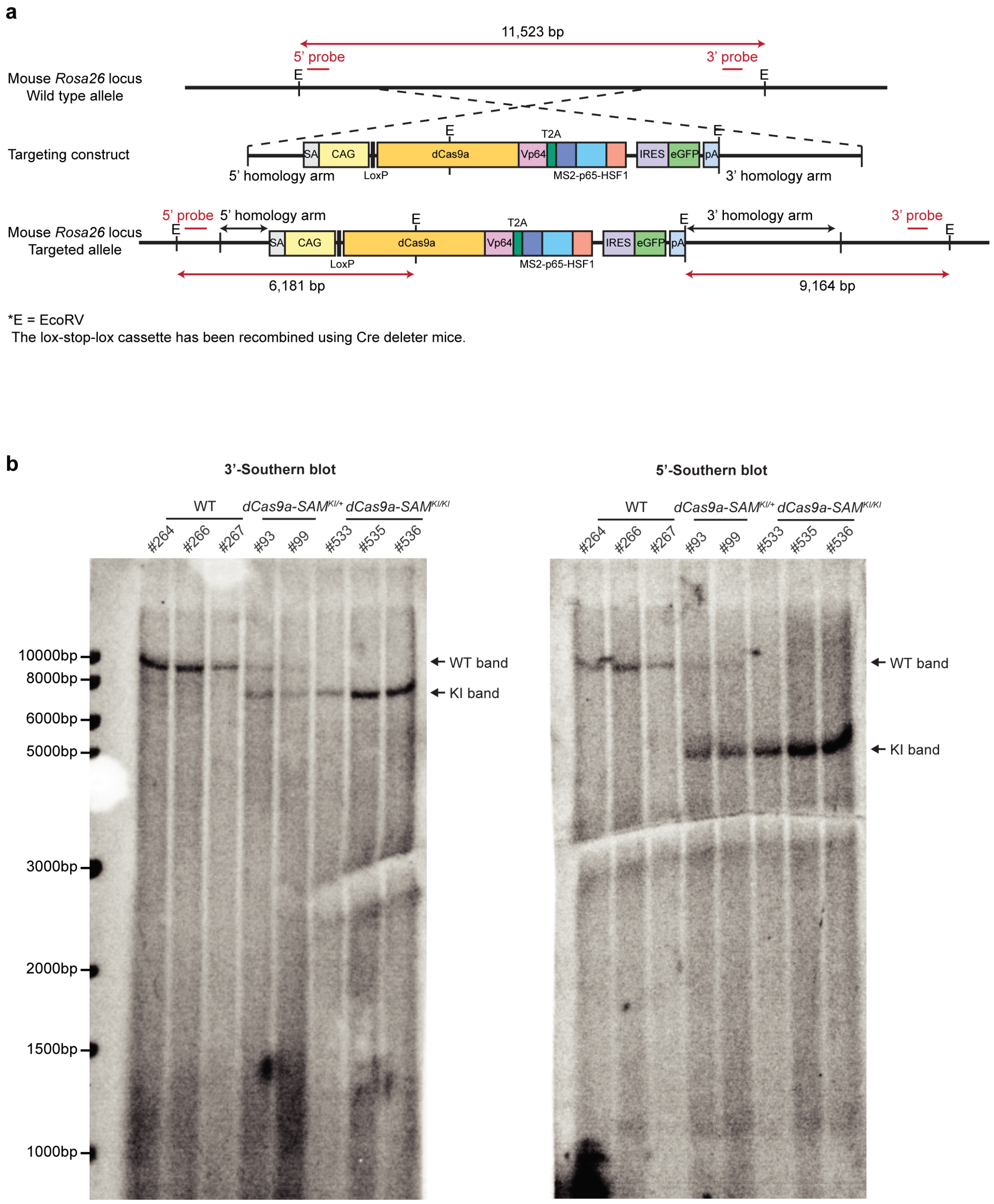

Supplementary Fig. 2 Southern blot analysis based validation of dCas9a-SAM cassette integration in gene-targeted mice. a Schematic representation of Southern blotting strategy. P32-labelled 3'- and 5'- *Rosa26* probes were used for the analysis. **b** Southern blot analysis for detecting WT or *dCas9a-SAM^{KI}* DNA bands. Genomic DNA samples used for Southern blotting were extracted from livers of WT, *dCas9a-SAM$^{KI/+}$* or *dCas9a-SAM$^{KI/KI}$* mice. 2 independent experiments were repeated with similar results. Source data are provided as a Source Data file.

Supplementary Fig. 3

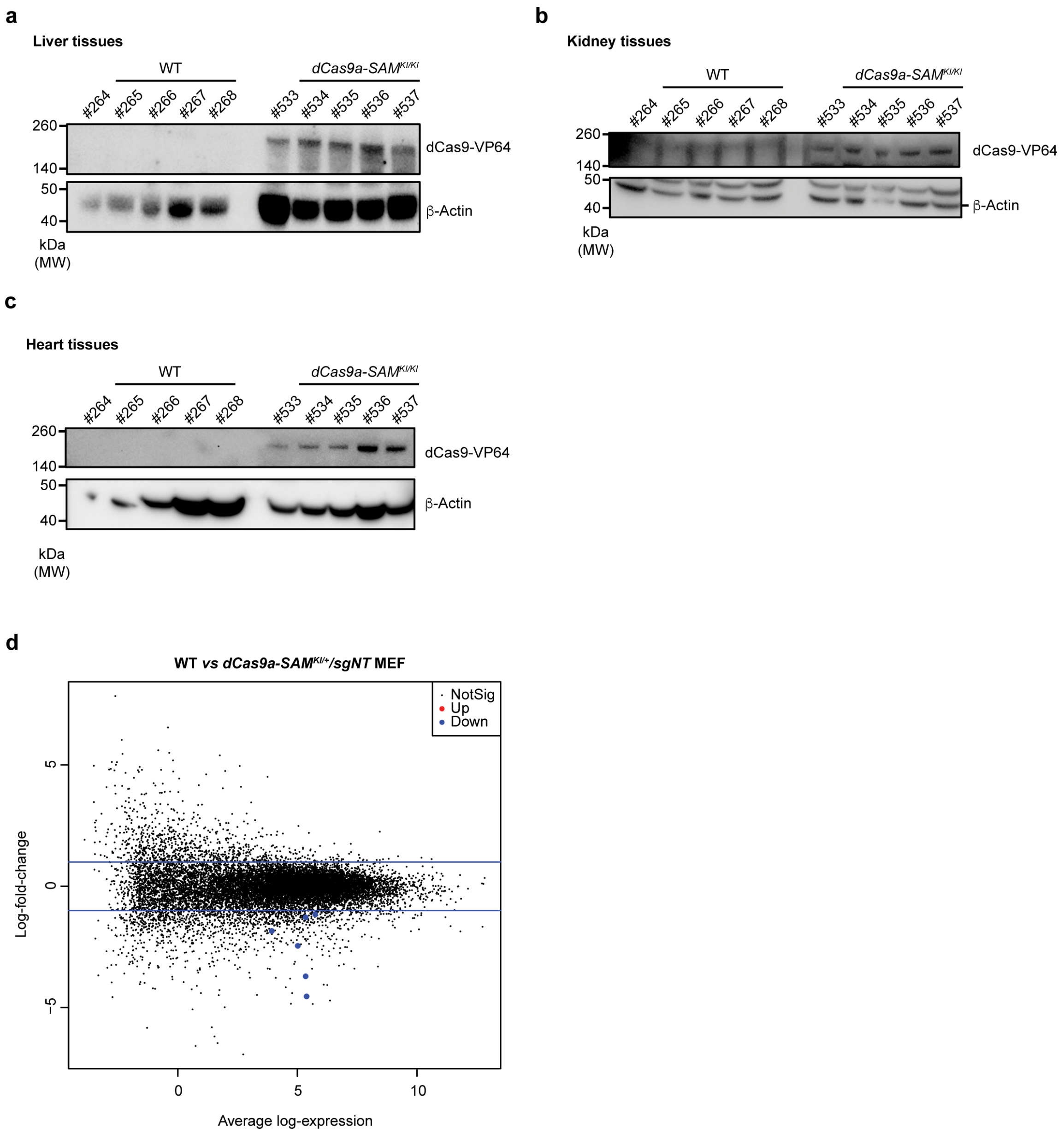

Supplementary Fig. 3 Tissue expression of dCas9-VP64 in *dCas9a-SAM* transgenic mice and analysis of off-target gene activation. a-c Western blot analysis for dCas9-VP64 fusion protein expression in liver and heart tissues harvested from WT or *dCas9a-SAM*$^{KI/KI}$ mice. Probing for β-Actin was used as a protein loading control. 2 independent experiments of each tissue were repeated with similar results. **d** Differential expression analysis of RNA from WT and *dCas9a-SAM*$^{KI/+}$*/sgNT* MEFs showing no significant changes in gene expression. For each genotype, 2 independent MEF cell lines were used for the analysis. Source data are provided as a Source Data file.

Supplementary Fig. 4

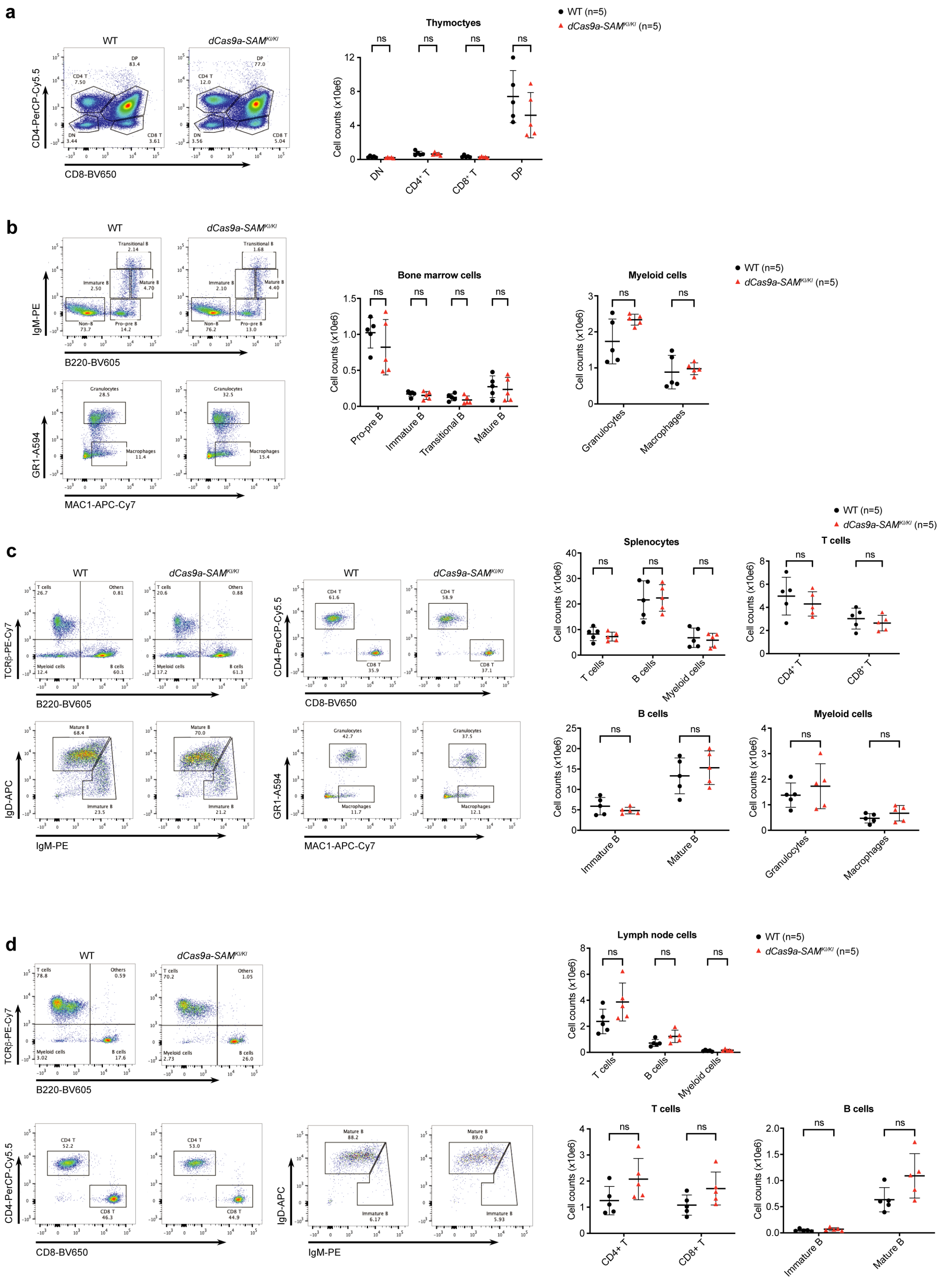

Supplementary Fig. 4 *dCas9a-SAM*$^{KI/KI}$ mice exhibit no defects in the haematopoietic system. a T cell composition in the thymi of WT mice or *dCas9a-SAM*$^{KI/KI}$ mice is shown by representative FACS plots. Distinct T cell populations were defined as DN (double negative, CD4^{-}

$CD8^{–}$) cells , $CD4^{+}$ T cells, $CD8^{+}$ T cells and DP (double positive, $CD4^{+}CD8^{+}$) T cells. **b** B cell and myeloid cell distributions in the bone marrow of WT mice or *dCas9a-SAM*$^{KI/KI}$ mice are shown by representative FACS plots. B cell subsets were defined as pro-B/pre-B ($B220^{+}IgM^{–}$), immature B ($B220^{+}IgM^{lo}$), transitional B ($B220^{+}IgM^{hi}$) and mature B ($B220^{hi}IgM^{lo}$) cells. Granulocytes were identified as $MAC1^{+}GR1^{+}$ and macrophages were identified as $MAC1^{+}GR1^{–}$. **c** T cell, B cell and myeloid cell distributions in the spleens of WT mice or *dCas9a-SAM*$^{KI/KI}$ mice are shown by representative FACS plots. In splenocytes, T cells were identified as $B220^{–}TCR\beta^{+}$, B cells as $B220^{+}TCR\beta^{–}$ and myeloid cells as $B220^{–}TCR\beta^{–}$. Specifically, T cell subsets were identified as $CD4^{+}$ T cells and $CD8^{+}$ T cells. B cell subsets were defined as immature B ($IgM^{+}IgD^{–}$) and mature B ($IgM^{–}IgD^{+}$) cells. Myeloid cell subsets were defined as granulocytes ($MAC1^{+}GR1^{+}$) and macrophages ($MAC1^{+}GR1^{–}$). **d** T cell, B cell and myeloid cell distributions in the lymph nodes of WT mice or *dCas9a-SAM*$^{KI/KI}$ mice are shown by representative FACS plots. In lymph node cells, T cells were defined as $B220^{–}TCR\beta^{+}$, B cells as $B220^{+}TCR\beta^{–}$ and myeloid cells as $B220^{–}TCR\beta^{–}$. T cell subsets were defined as $CD4^{+}$ T cells and $CD8^{+}$ T cells. B cell subsets were defined as immature B ($IgM^{+}IgD^{–}$) and mature ($IgM^{–}IgD^{+}$) B cells. Data are presented as mean ± SD, n=5 mice for each group. All the statistical significance was determined using multiple unpaired *t*-test with two-stage step-up correction. ns = no significant difference. Source data are provided as a Source Data file.

Supplementary Fig. 5

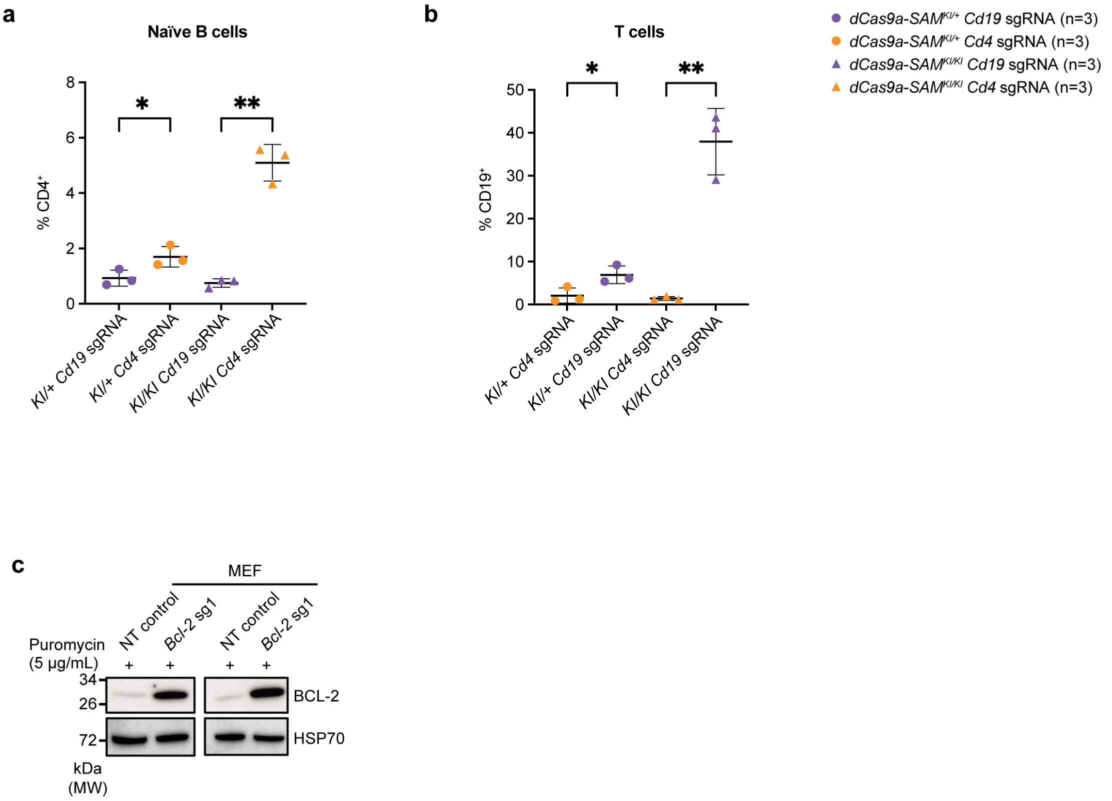

Supplementary Fig. 5 Comparison of robust gene expression in *dCas9a-SAM*$^{KI/+}$ and *dCas9a-SAM*$^{KI/KI}$ primary murine cells. a Naïve B cells were isolated from *dCas9a-SAM*$^{KI/+}$ or *dCas9a-SAM*$^{KI/KI}$ mice and transduced with *Cd4* sgRNAs. The expression of CD4 was analysed by flow cytometry. Cells transduced with *Cd19* sgRNAs served as the negative control for CD4 staining. Data are presented as mean ± SD, n = 3 independent experiments. The statistical significance was determined by two-sided student's *t*-test. $P = 0.0465$ (*KI/+ Cd4 vs KI/+ Cd19*), $P = 0.0004$ (*KI/KI Cd4 vs KI/KI Cd19*), * = $P < 0.05$, ** = $P < 0.01$. **b** T cells were isolated from *dCas9a-SAM*$^{KI/+}$ or *dCas9a-SAM*$^{KI/KI}$ mice and transduced with *Cd19* sgRNAs. The expression of CD19 was analysed by flow cytometry. Cells transduced with *Cd4* sgRNAs served as the negative control. Data are presented as mean ± SD, n = 3 independent experiments. The statistical significance was determined by two-sided student's *t*-test. $P = 0.0375$ (*KI/+ Cd19 vs KI/+ Cd4*), $P = 0.0012$ (*KI/KI Cd19 vs KI/KI Cd4*), * = $P < 0.05$, ** = $P < 0.01$. **c** MEFs were generated from E14 embryos of *dCas9a-SAM*$^{KI/+}$ mice and transduced with *Bcl-2* sgRNAs or non-targeting control sgRNAs. sgRNAs in puromycin-resistant backbones were used for Western blotting. Probing for HSP70 served as a loading control. 2 independent experiments were repeated with similar results. Source data are provided as a Source Data file.

Supplementary Fig. 6

a

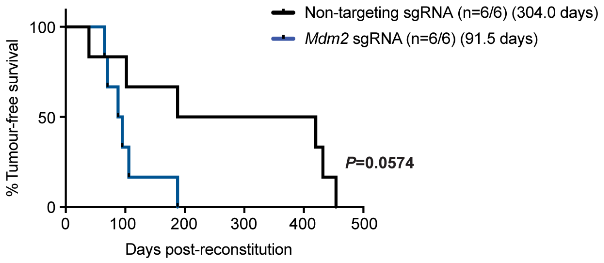

b

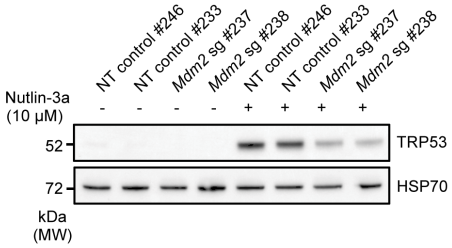

Supplementary Fig. 6 Haematopoietic reconstitution with *Eμ-Myc/dCas9a-SAM$^{KI/+}$/sgMdm2* HSPCs. a Kaplan-Meier survival curve of reconstituted mice transplanted with non-targeting controls or *Eμ-Myc/dCas9a-SAM$^{KI/+}$/sgMdm2* HSPCs. n/n = numbers of sick mice/numbers of total recipient mice. Data are combined from two independent reconstitution experiments. The statistical significance was determined by the Mantel-Cox test. P = 0.0574. **b** The expression of TRP53 in control lymphoma-derived cell lines or *Eμ-Myc/dCas9a-SAM$^{KI/+}$/sgMdm2* lymphoma-derived cell lines were determined by Western blot analysis. Cell lines were pre-treated with 20 μM Q-VD-OpH for 15 min and then treated with DMSO or 10 μM nutlin-3a for 24 h. Cell line numbers indicate the animal numbers of sick mice from which the cell lines had been derived. Probing for HSP70 served as a protein loading control. 2 independent experiments were repeated with similar results. Source data are provided as a Source Data file.

Supplementary Fig. 7

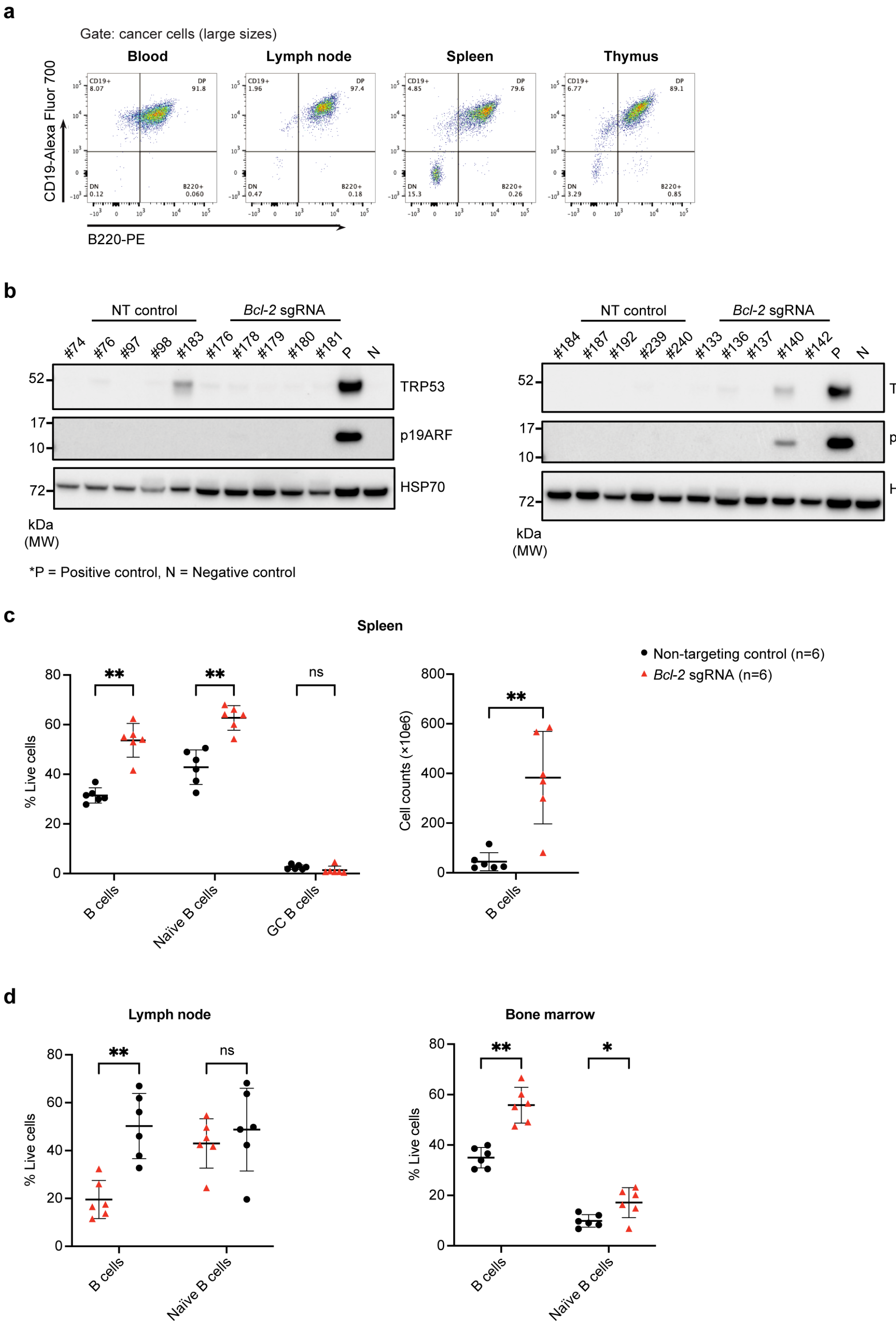

Supplementary Fig. 7 Haematopoietic reconstitution with *Eμ-Myc/dCas9a-SAM$^{KI/+}$/sgBcl-2* HSPCs. a Characterisation of *Eμ-Myc/dCas9a-SAM$^{KI/+}$/sgBcl-2* lymphoma phenotype. Blood, lymph nodes, spleen and thymus were harvested from sick *sgBcl-2* HSPC reconstituted mice for staining and flow cytometric analysis. Lymphoma cells were gated on FSC/SSC at a larger size than non-transformed haematopoietic cells. **b** TRP53 and p19ARF protein analysis in the spleens

from sick control HSPC or *sgBcl-2* HSPC reconstitued mice is shown by Western blotting. Probing for HSP70 served as a protein loading control. 2 independent experiments were repeated with similar results. **c, d** Lethally irradiated mice were transplanted with *Eμ-Myc/dCas9a-SAM*$^{KI/+}$*/sgNT* (control) or *Eμ-Myc/dCas9a-SAM*$^{KI/+}$*/sgBcl-2* HSPCs. Pre-leukemic analysis was performed 5 weeks post HSPC transplantation. B cells were defined as B220^{+}CD19^{+}. Naïve B cells and GC B cells were gated from B220^{+}CD19^{+} cells and defined as naïve B (CD38^{+}FAS$^{–}$) or GC B (CD38$^{–}$FAS^{+}) cells in **c** spleen and **d** lymph node and bone marrow. Data are presented as mean ± SD, n=6 mice for each group. The statistical significance was determined using multiple unpaired *t*-test with two-stage step-up correction. In **c** spleen (left panel), P < 0.0001 (B cells), P = 0.0002 (Naïve B cells), P = 0.1371 (GC B cells). In **d** lymph node, P = 0.0008 (B cells), P = 0.5012 (Naïve B cells). In **d** bone marrow, P < 0.0001 (B cells), P = 0.0203 (Naïve B cells). In **c** spleen (right panel) , the statistical significance was determined using two-sided student's *t*-test, P = 0.0014. ns = no significant difference, * = P < 0.05, ** = P < 0.01. Source data are provided as a Source Data file.

Supplementary Fig. 8

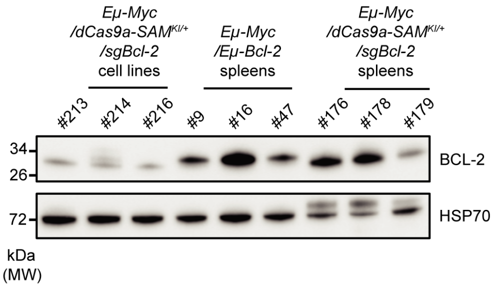

Supplementary Fig. 8 Comparison of BCL-2 levels in lymphomas from *Eμ-Myc/Eμ-Bcl-2* doubly transgenic mice and lymphomas from *Eμ-Myc/dCas9a-SAM$^{KI/+}$/sgBcl-2* HSPC reconstitued mice. Western blot analysis for BCL-2 in *Eμ-Myc/dCas9a-SAM$^{KI/+}$/sgBcl-2* cell lines (lanes 1-3) and primary tumours (lanes 7-9) and from *Eμ-Myc/Eμ-Bcl-2* primary tumours (lanes 4-6) using an antibody that recognises both mouse and human BCL-2. Probing for HSP70 served as a loading control. 2 independent experiments were repeated with similar results. Source data are provided as a Source Data file.

Supplementary Fig. 9

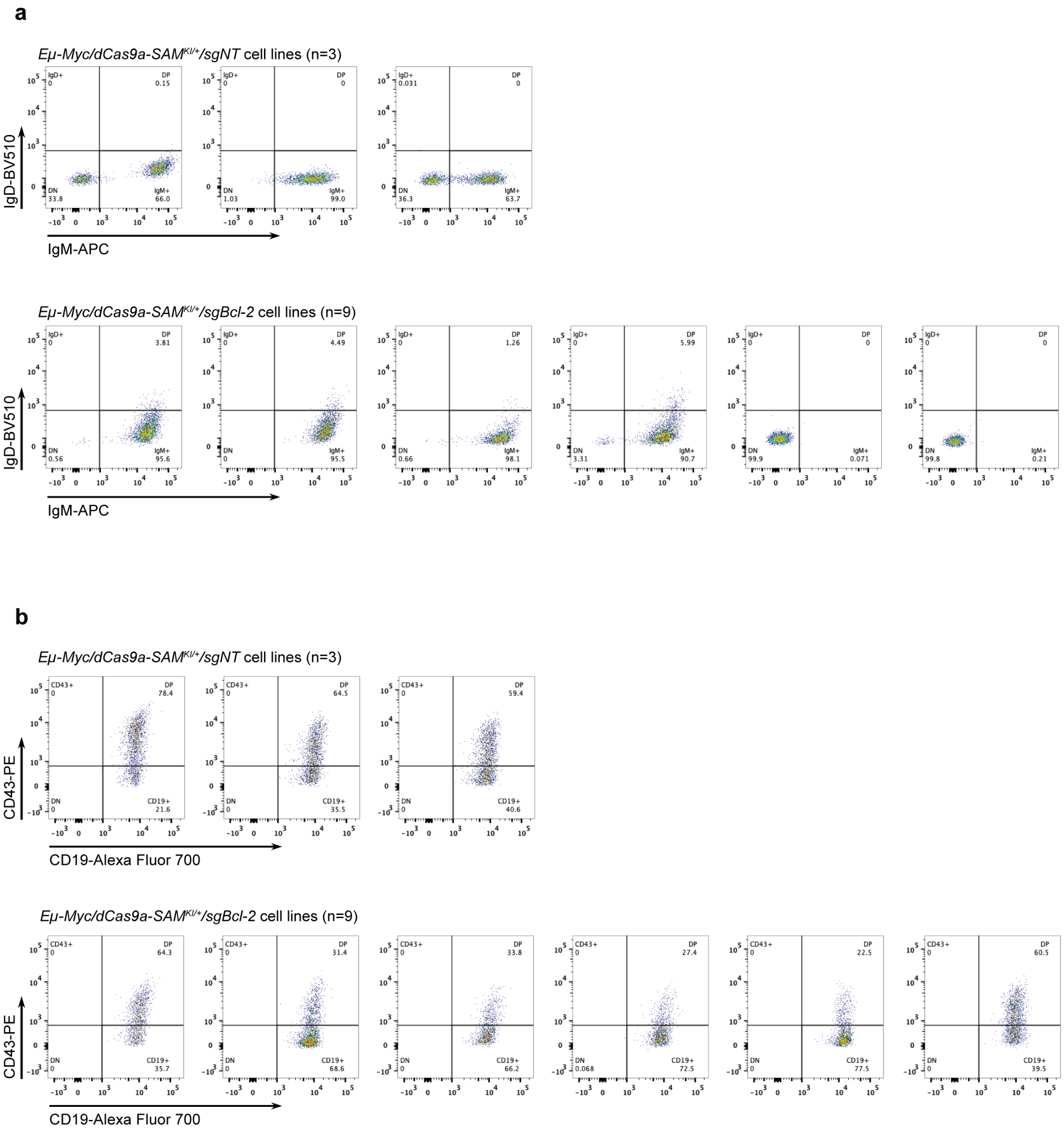

Supplementary Fig. 9 Phenotyping of *Eμ-Myc/dCas9a-SAM$^{KI/+}$/sgBcl-2* lymphoma-derived cell lines. a, b IgM/IgD or CD19/CD43 staining was performed and analysed by flow cytometric analysis. 3 control cell lines and 7 *Eμ-Myc/dCas9a-SAM$^{KI/+}$/sgBcl-2* lymphoma cell lines were used for the analysis and all FACS plots are shown respectively.

Supplementary Fig. 10

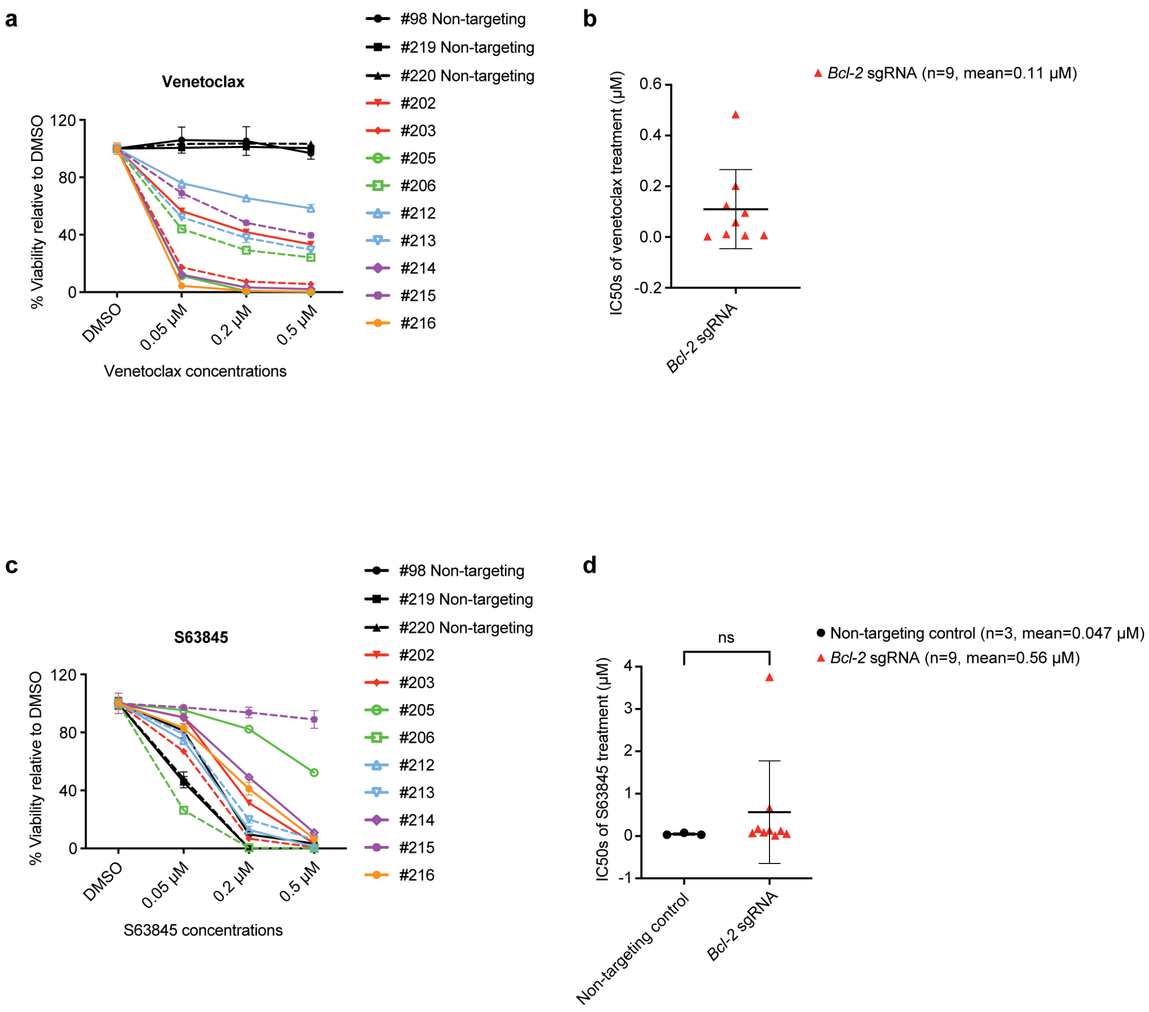

Supplementary Fig. 10 Responses of lymphoma cell lines to treatment with the BCL-2 inhibitor venetoclax or the MCL-1 inhibitor S63845. a, c Viability curves of distinct control *Eμ-Myc* lymphoma cell lines (n=3) or *Eμ-Myc/dCas9a-SAM*$^{KI/+}$*/sgBcl-2* lymphoma cell lines (n=9) after treatment for 24 h with the indicated doses of the BCL-2 inhibitor venetoclax or the MCL-1 inhibitor S63845. Cell viability was determined by staining with propidium iodide (PI) followed by flow cytometric analysis. Data are presented as mean ± SD, n = 3 independent experiments. **b, d** IC50s of individual control *Eμ-Myc* lymphoma lines or *Eμ-Myc/dCas9a-SAM*$^{KI/+}$*/sgBcl-2* lymphoma lines treated with venetoclax or S63845. Control *Eμ-Myc* lymphoma cell lines are resistant to venetoclax treatment and so the IC50s cannot be calculated and are therefore not presented in the figure. Data are represented as mean ± SD, 3 control cell lines and 9 *Eμ-Myc/dCas9a-SAM*$^{KI/+}$*/sgBcl-2* lymphoma cell lines were used for each treatment. The statistical significance was determined using two-sided Mann-Whitney *t*-test. ns = no significant difference. Source data are provided as a Source Data file.

Supplementary Fig. 11

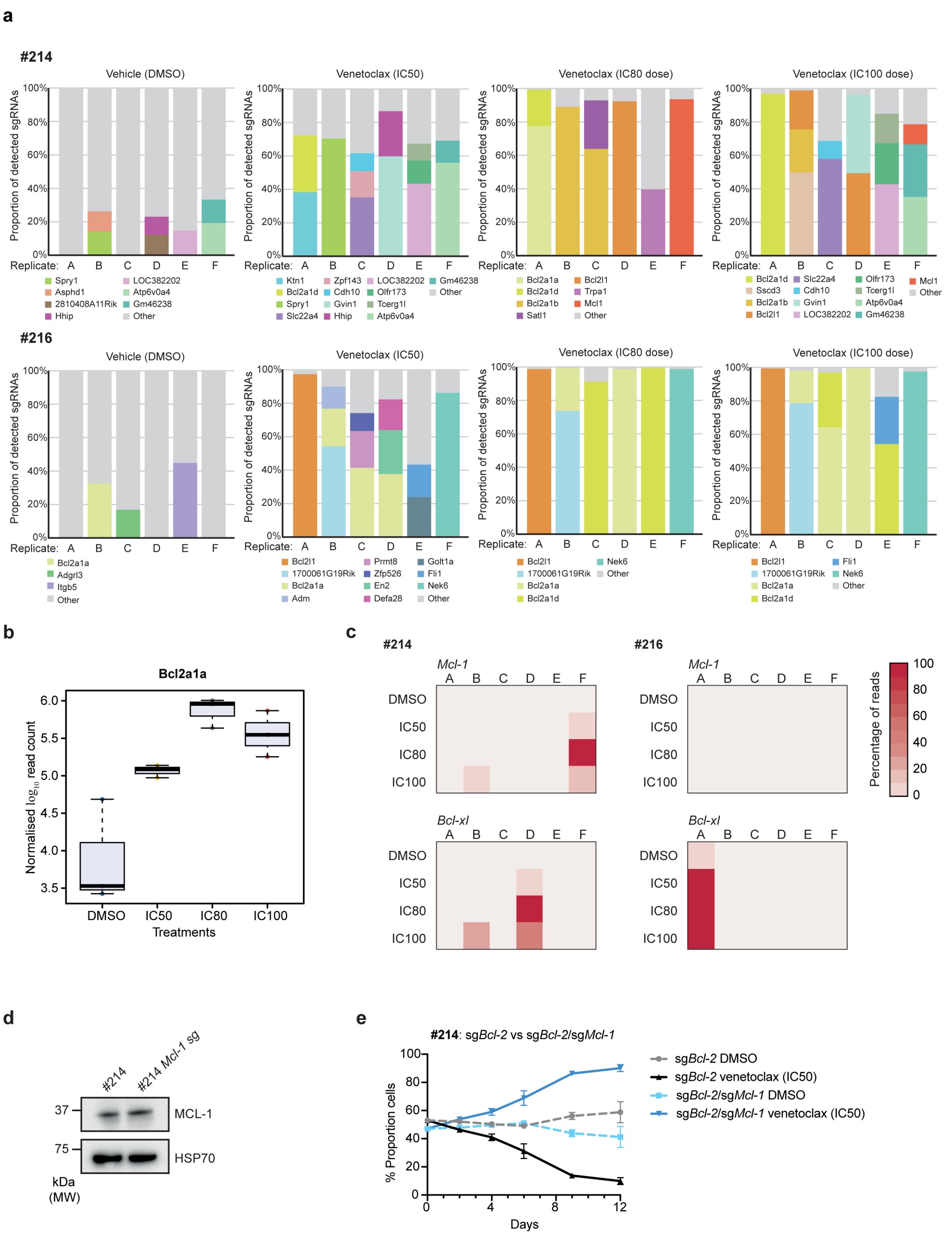

Supplementary Fig. 11 Whole genome CRISPR activation screens for venetoclax resistance factors in *Eµ-Myc/dCas9a-SAM$^{KI/+}$/sgBcl-2* lymphoma cell lines. a Top enriched sgRNAs for each replicate in each treatment group. sgRNAs making up at least 10% of the total sample are

indicated. **b** Representative box plot showing enrichment of sgRNAs targeting the promoter of *Bcl2a1a* in #216 cells treated with various doses of venetoclax compared to control cells treated with DMSO. Data are presented as mean ± SD, n = 6 independent cell samples. The minima, maxima, centre, bounds of box and whiskers and percentile are provided in the Source Data file. **c** Heatmaps showing the proportions of sequencing read results which mapped to sgRNAs targeting the promoter regions of the pro-survival genes *Mcl1* or *Bcl-xl*. The six replicate samples for each treatment condition are shown in columns A-F. **d** Western blot analysis for MCL-1 in the #214 lymphoma-derived cell line with and without an sgRNA targeting the *MCL-1* promoter (sg*Mcl-1*). 2 independent experiments were repeated with similar results. **e** Cell competition assays of #214 control DHL-like cells *vs* the same cell lines carrying an additional sgRNA targeting *Mcl-1* (sg*Mcl-1*) as shown in d. Lymphoma cells were mixed ~1:1 and treated with DMSO (negative control) or IC50 doses of venetoclax. Parental lymphoma cells were tagged with eGFP and the contribution of lymphoma cells of each genotype to the overall population was monitored over time by flow cytometry. Data are presented as mean ± SD with representative graph showing one of three experiments performed in duplicate of similar results. Source data are provided as a Source Data file.

Supplementary Fig. 12

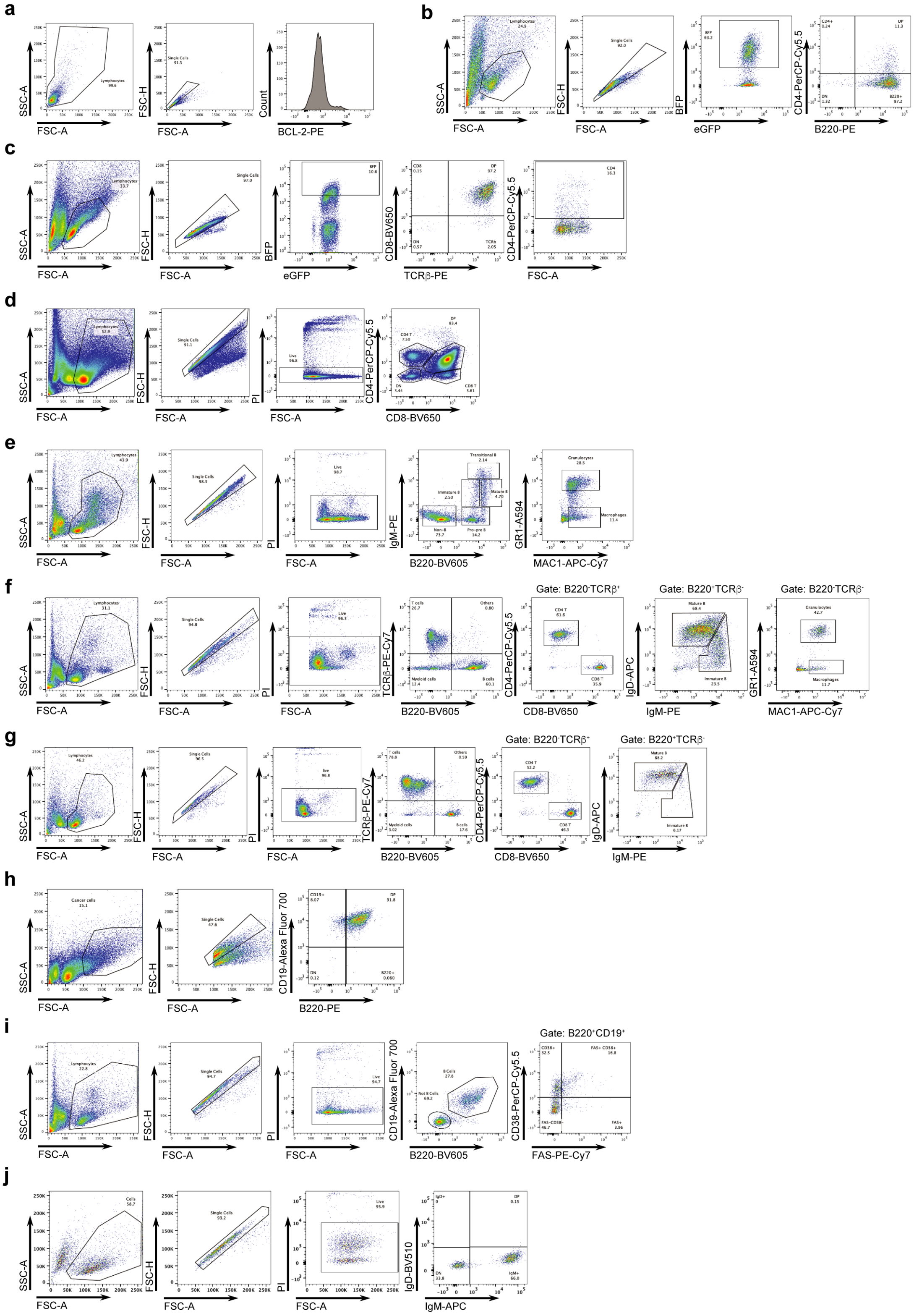

Supplementary Fig. 12 FACS analytical strategies. a Representative FACS plots show the gating strategy for intracellular staining of Fig. 2b, Fig. 3a, Fig. 4b, Fig. 5b. **b** Representative FACS plots show the gating strategy for naïve B cell staining of Fig. 3b-c, Fig. 3f. **c** Representative FACS

plots show the gating strategy for T cell staining of Fig. 3d-e. **d** Representative FACS plots show the gating strategy for thymocytes of Supplementary Fig. 4a. **e** Representative FACS plots show the gating strategy for bone marrow cells of Supplementary Fig. 4b. **f** Representative FACS plots show the gating strategy for splenocytes of Supplementary Fig. 4c. **g** Representative FACS plots show the gating strategy for lymph node cells of Supplementary Fig. 4d. **h** Representative FACS plots show the gating strategy for cancer cells from tumour tissues of Supplementary Fig. 7a. **i** Representative FACS plots show the gating strategy for pre-leukaemic analysis of Supplementary Fig. 7c-d. **j** Representative FACS plots show the gating strategy for *Eμ-Myc/dCas9a-SAM*$^{KI/+}$*/sgNT* or *sgBcl2* cell lines of Supplementary Fig. 9a-b. The gating strategy of Fig. 2c is the same as live cell gating in Supplementary Fig. 12d-g. The gating strategy of Supplementary Fig. 1a is the same as live cell gating in Supplementary Fig. 12j.

Supplementary Table 1. IC50s of *Eμ-Myc/dCas9a/SAM$^{KI/+}$/sgBcl-2* lymphoma cell lines or control *Eμ-Myc* lymphoma cell lines for treatment with venetoclax or S63845

Sample name	IC50 venetoclax (μM)	Sample name	IC50 S63845 (μM)
#98 Non-targeting	Resistant	#98 Non-targeting	0.0306
#219 Non-targeting	Resistant	#219 Non-targeting	0.0329
#220 Non-targeting	Resistant	#220 Non-targeting	0.0775
#202	0.1237	#202	0.1213
#203	0.0114	#203	0.0565
#205	0.0060	#205	0.6606
#206	0.0585	#206	0.0153
#212	0.4836	#212	0.0710
#213	0.0957	#213	0.0887
#214	0.0070	#214	0.1735
#215	0.2017	#215	3.7590
#216	0.0023	#216	0.1329

Supplementary Table 2. Genetic aberrations associated with DHL-BCL-2 in *Eμ-Myc/dCas9a-SAM*$^{KI/+}$*/sgBcl-2* lymphoma cell line *vs* preB-ALL samples

Symbol	logFC	AveExpr	t	*P* Value	adj. *P* Val	B
Bcl2	3.03189704	6.47865835	10.767508	2.85E-06	9.72E-05	5.25618765
Myc	2.77160562	10.1998518	7.6344228	4.35E-05	0.00058838	2.16180242
Ccnd3	-2.7920331	7.6830191	-4.9339313	0.00101976	0.00524665	-1.2170688
Ep300	0.68572877	7.17750639	4.3817201	0.00198561	0.00840557	-1.9372383
Id3	-1.2449087	6.33699475	-3.2608508	0.01060075	0.02904999	-3.6985044
Bcl6	-2.6223456	0.56892865	-2.4145289	0.04067919	0.08353911	-4.2020396
Trp53	-0.3153439	7.97362703	-2.1427842	0.06224146	0.1162064	-5.5029926
Kras	0.48356277	6.51391025	1.76100632	0.11375822	0.18798887	-6.0352654
Card11	0.51811362	5.80516733	1.18387129	0.26856595	0.36892007	-6.7151314
Braf	-0.2389598	5.30368528	-1.0908689	0.30504277	0.40824343	-6.8100652
Stat6	0.19541311	7.10817456	0.97207585	0.35764185	0.46252276	-7.0400542
Ezh2	0.15396148	7.90584374	0.66625792	0.52277057	0.61981593	-7.3062096
Crebbp	-0.0208034	5.94473496	-0.081113	0.93721426	0.95422244	-7.4857799
Tcf3	-0.0224663	9.26224201	-0.0719752	0.94427287	0.9600356	-7.5513546

The statistical significance between groups was determined by one-sided moderated *t*-test and multiple comparisons were adjusted to control the false discovery rate (FDR) using the method of Benjamini and Hochberg.

Supplementary Table 3. Guide RNAs designed for CRISPR activation system

Targets	gRNA sequences (5'→3')
Non-targeting	GCCGTAAGCGGGCCGGTTGA
Bcl-2 sgRNA1	GCGGGCGGGCGCTCAGAGGA
Bcl-2 sgRNA2	CAGGAAACCAGGCGCTCCGG
Bcl-2 sgRNA3	GGAGAATGAAGTAAGAGGCC
Cd4 sgRNA	TCTGGAATGTCACTATTGTT
Cd19 sgRNA	TAAGTGCTGGGTGACAGGGA
Irf4 sgRNA	CTGCGGGACACCCAGGCACA
Mdm2 sgRNA	CGCGCGCACGCCCCGATGCC
Bcl2a1a (A1) sgRNA	AGCCAAAGTTCAGACTGTGA
Mcl-1 sgRNA	TTCCGGCCGGAGCGCTCTCG

Supplementary Table 4. Detailed information on mice used for haematopoietic cell analysis

Sample name	Strain	Gender	Age in days
#264	WT	Male	60
#265	WT	Male	60
#266	WT	Male	60
#267	WT	Male	60
#268	WT	Male	60
#533	*dCas9a-SAM*$^{KI/KI}$	Male	64
#534	*dCas9a-SAM*$^{KI/KI}$	Male	64
#535	*dCas9a-SAM*$^{KI/KI}$	Male	64
#536	*dCas9a-SAM*$^{KI/KI}$	Male	64
#537	*dCas9a-SAM*$^{KI/KI}$	Male	64

Supplementary Table 5. Fluorochrome conjugated antibodies used for immune cell staining

Antibody	Dilutions	Clone name	Catalog number	Source
B220	1:200	RA3-6B2	#103244	BioLegend
CD4	1:800	Gk1.5	#100434	BioLegend
CD8	1:400	53-6.7	#563234	BD Horizon
IgD	1:400	11-26c.2a	#563110	BD Horizon
IgM	1:50	5.1	N/A (in house)	WEHI
MAC1	1:800	M1/70	#557657	BD Pharmingen
GR1	1:400	RB6-8C5	#108448	BioLegend
TCRβ	1:400	H57-597	#109222	BioLegend
CD19	1:400	1D3	#152414	BioLegend
TER-119	1:200	TER-119	#553672	BD Pharmingen
CD138	1:400	281-2	#561070	BD Pharmingen
CD38	1:500	Ab90	#562770	BD Pharmingen
FAS	1:200	Jo2	#557653	BD Pharmingen
CD43	1:200	S7	N/A (in house)	WEHI
Cas9 (for Intracellular staining)	1:50	7A9-3A3	#35193	Cell Signaling Technology
BCL-2 (for Intracellular staining)	1:100	BCL2/10C4	#633508	BioLegend
BIM (for Intracellular staining)	1:100	3C5	N/A (in house)	WEHI

Supplementary Table 6. Primary antibodies used for Western blot analysis

Antibody	Dilutions	Clone name	Catalog number	Source
HSP70	1:10000	N6	N/A (in house)	Gift, Dr W. Welch, USCF
β-Actin	1:1000	13E5	#4970	Cell Signaling Technology
Mouse BCL-2	1:1000	3F11	#554218	BD Pharmingen
Human/mouse BCL-2	1:1000	7/Bcl-2	#610539	BD Transduction Laboratories
TRP53	1:500	CM5	#NCL-L-p53-CM5p	Novocastra
BIM	1:1000	ploycolone	#ADI-AAP-330	Enzo
MCL-1	1:1000	14C11-20	N/A (in house)	Gift, DCS Huang, WEHI
BCL-XL	1:1000	9C9	N/A (in house)	WEHI
A1	1:500	6D6	N/A (in house)	WEHI
