# Supplementary material for: Generation of a CRISPR activation mouse that enables modelling of aggressive lymphoma and interrogation of venetoclax resistance
Source: Nat Commun. 2022 Aug 12;13:4739. doi: 10.1038/s41467-022-32485-9 (PMC9374748; doi:10.1038/s41467-022-32485-9)
Supplement: Supplementary file 3 — Description of Additional Supplementary Files [file 41467_2022_32485_MOESM3_ESM.pdf]

Supplementary Data 1: Molecular Signature database analysis *Eμ-Myc/dCas9a-SAM*$^{KI/+}$*/sgBcl-2* lymphomas *vs* preB-ALL.

Supplementary Data 2: Significantly enriched sgRNAs from whole genome CRISPR activation screens in *Eμ-Myc/dCas9a-SAM*$^{KI/+}$*/sgBcl-2* cell line #214, #216 treated with IC80 doses of venetoclax *vs* treatment with (negative control) DMSO.

Supplementary Data 3: CRISPR screen sequencing read raw data of cell line #214, #216.
