# Supplementary material for: Generation of a CRISPR activation mouse that enables modelling of aggressive lymphoma and interrogation of venetoclax resistance
Source: Nat Commun. 2022 Aug 12;13:4739. doi: 10.1038/s41467-022-32485-9 (PMC9374748; doi:10.1038/s41467-022-32485-9)
Supplement: Supplementary file 7 — Reporting Summary [file 41467_2022_32485_MOESM7_ESM.pdf]

nature portfolio

Corresponding author(s): Marco Herold

Last updated by author(s): Jul 16, 2022

Reporting Summary

Nature Portfolio wishes to improve the reproducibility of the work that we publish. This form provides structure for consistency and transparency in reporting. For further information on Nature Portfolio policies, see our Editorial Policies and the Editorial Policy Checklist.

Statistics

For all statistical analyses, confirm that the following items are present in the figure legend, table legend, main text, or Methods section.

n/a	Confirmed	
☐	☒	The exact sample size (*n*) for each experimental group/condition, given as a discrete number and unit of measurement
☐	☒	A statement on whether measurements were taken from distinct samples or whether the same sample was measured repeatedly
☐	☒	The statistical test(s) used AND whether they are one- or two-sided<br>*Only common tests should be described solely by name; describe more complex techniques in the Methods section.*
☒	☐	A description of all covariates tested
☐	☒	A description of any assumptions or corrections, such as tests of normality and adjustment for multiple comparisons
☐	☒	A full description of the statistical parameters including central tendency (e.g. means) or other basic estimates (e.g. regression coefficient) AND variation (e.g. standard deviation) or associated estimates of uncertainty (e.g. confidence intervals)
☐	☒	For null hypothesis testing, the test statistic (e.g. *F*, *t*, *r*) with confidence intervals, effect sizes, degrees of freedom and *P* value noted<br>*Give P values as exact values whenever suitable.*
☒	☐	For Bayesian analysis, information on the choice of priors and Markov chain Monte Carlo settings
☒	☐	For hierarchical and complex designs, identification of the appropriate level for tests and full reporting of outcomes
☒	☐	Estimates of effect sizes (e.g. Cohen's *d*, Pearson's *r*), indicating how they were calculated

Our web collection on statistics for biologists contains articles on many of the points above.

Software and code

Policy information about availability of computer code

Data collection	Flow cytometry data was acquired using BD FACSDiva software v8.0.
Data analysis	Flow cytometry data was analysed with FlowJo v10. Graphs were generated and P-values were calculated with Prism v8.2.0. RNA sequencing data was analysed and visualized with Rsubread v2.6.4, edgeR v3.34.1, limma v3.48.3, pheatmap v1.0.12, Molecular Signatures Database v7.5.1, and MiXCR v3.0.6 software. CRISPRa screening data was analysed with edgeR v3.30.3 software and relevant custom codes are deposited in the GitHub [https://github.com/goknurginer/crispra-screen-analysis].

For manuscripts utilizing custom algorithms or software that are central to the research but not yet described in published literature, software must be made available to editors and reviewers. We strongly encourage code deposition in a community repository (e.g. GitHub). See the Nature Portfolio guidelines for submitting code & software for further information.

Data

Policy information about availability of data

All manuscripts must include a data availability statement. This statement should provide the following information, where applicable:

- Accession codes, unique identifiers, or web links for publicly available datasets
- A description of any restrictions on data availability
- For clinical datasets or third party data, please ensure that the statement adheres to our policy

RNA-seq data generated in this study have been deposited in the NCBI Gene Expression Omnibus (GEO) database under accession code GSE205509 [https://

www.ncbi.nlm.nih.gov/geo/query/acc.cgi?acc=GSE205509]. The Molecular Signatures Database used in this study is available online [https://www.gsea-msigdb.org/gsea/msigdb]. Source data are provided with this paper.

Human research participants

Policy information about studies involving human research participants and Sex and Gender in Research.

Reporting on sex and gender	Not applicable.
Population characteristics	Not applicable.
Recruitment	Not applicable.
Ethics oversight	Not applicable.

Note that full information on the approval of the study protocol must also be provided in the manuscript.

Field-specific reporting

Please select the one below that is the best fit for your research. If you are not sure, read the appropriate sections before making your selection.

☒ Life sciences ☐ Behavioural & social sciences ☐ Ecological, evolutionary & environmental sciences

For a reference copy of the document with all sections, see nature.com/documents/nr-reporting-summary-flat.pdf

Life sciences study design

All studies must disclose on these points even when the disclosure is negative.

Sample size	No sample size calculations were performed but sample sizes were chosen based on researchers' experience and widely used numbers of experimental replicates in the published literature. Sample sizes were also determined according to enable statistical analyses . For in vitro experiments, 2-9 independent biological cell lines were used for at least two independent experiments (some of experiments only used 2 individual cell lines because the variability between biological repeats was very low). For in vivo experiments, for each experiment at least 3 to 5 mice per group were used and experiments were performed twice to ensure the reproducibility. The chosen sample size was sufficient to determine statistical significance in our experiments.
Data exclusions	No data was excluded from the analysis.
Replication	All experiments were performed at least two or three times with similar results.
Randomization	Mice were grouped according to the genotype. Randomization was used for lethally irradiated C57BL/6-Ly5.1+ mice to perform HSPCs injections. For each experiment mice were age- and sex-matched.
Blinding	The animal technicians looking after the mice and making the call on their sickness (lymphoma) were blinded to the nature of the experiment.

Reporting for specific materials, systems and methods

We require information from authors about some types of materials, experimental systems and methods used in many studies. Here, indicate whether each material, system or method listed is relevant to your study. If you are not sure if a list item applies to your research, read the appropriate section before selecting a response.

Materials & experimental systems

n/a	Involved in the study	
☐	☒	Antibodies
☐	☒	Eukaryotic cell lines
☒	☐	Palaeontology and archaeology
☐	☒	Animals and other organisms
☒	☐	Clinical data
☒	☐	Dual use research of concern

Methods

n/a	Involved in the study	
☒	☐	ChIP-seq
☐	☒	Flow cytometry
☒	☐	MRI-based neuroimaging

Antibodies

Antibodies used

Antibodies used for flow cytometry were:
B220 (RA3-6B2, #103244, BioLegend)
CD4 (Gk1.5, #100434, BioLegend)
CD8 (53-6.7, #563234, BD Horizon)
IgD (11-26c.2a, #563110, BD Horizon)
IgM (5.1, WEHI)
MAC1 (M1/70, #557657, BD Pharmingen)
GR1 (RB6-8C5, #108448, BioLegend)
TCRbeta (H57-597, #109222, BioLegend)
CD19 (1D3, #152414, BioLegend)
TER-119 (TER-119, #553672, BD Pharmingen)
CD138 (281-2, #561070, BD Pharmingen)
CD38 (Ab90, #562770, BD Pharmingen)
FAS (Jo2, #557653, BD Pharmingen)
CD43 (S7, WEHI)
Cas9 (7A9-3A3, #35193, Cell Signaling Technology)
BCL-2 (BCL2/10C4, #633508, BioLegend)
BIM (3C5, WEHI)

Primary antibodies used for Western blotting were:
HSP70 (N6, Gift, Dr W. Welch, USCF)
beta-Actin (13E5, #4970, Cell Signaling Technology)
Mouse BCL-2 (3F11, #554218, BD Pharmingen)
Human/mouse BCL-2 (7/Bcl-2, #610539, BD Transduction Laboratories)
TRP53 (CM5, #NCL-L-p53-CM5p, Novocastra)
BIM (polyclone, #ADI-AAP-330, Enzo)
MCL-1 (14C11-20, Gift, DCS Huang, WEHI)
BCL-XL (9C9, WEHI)
A1 (6D6, WEHI)

Secondary antibodies used for Western blotting were:
HRP-conjugated secondary antibodies to detect mouse IgG (#1010-05, polyclonal, Southern Biotech)
HRP-conjugated secondary antibodies to detect rat IgG (#3010-05, polyclonal, Southern Biotech)
HRP-conjugated secondary antibodies to detect rabbit IgG (#4010-05, polyclonal, Southern Biotech)

Validation

Validation data for commercial antibodies are available on vendor websites. Flow cytometry antibodies were validated by demonstrating that they stain the cell types that express the surface markers detected by these antibodies. Antibodies for Western blotting were validated by showing that they recognize a protein of the expected molecular weight and that the intensity of the protein band is increased when cells are manipulated to overexpress targeting corresponding genes. Detailed validation information was as followed:

Antibodies used for flow cytometry:
B220 (RA3-6B2, BioLegend) https://www.biolegend.com/nl-be/products/brilliant-violet-605-anti-mouse-human-cd45r-b220-antibody-7870
CD4 (Gk1.5, BioLegend) https://www.biolegend.com/fr-ch/products/percp-cyanine5-5-anti-mouse-cd4-antibody-4220
CD8 (53-6.7, BD Horizon) https://www.bdbiosciences.com/en-au/products/reagents/flow-cytometry-reagents/research-reagents/single-color-antibodies-ruo/bv650-rat-anti-mouse-cd8a.563234
IgD (11-26c.2a, BD Horizon) https://www.bdbiosciences.com/en-au/products/reagents/flow-cytometry-reagents/research-reagents/single-color-antibodies-ruo/bv510-rat-anti-mouse-igd.563110
IgM (5.1, WEHI) This antibody was validated by demonstrating that it could distinguish B cell populations that expressed IgM or not (Supp. Fig. 4b).
MAC1 (M1/70, BD Pharmingen) https://www.bdbiosciences.com/en-au/products/reagents/flow-cytometry-reagents/research-reagents/single-color-antibodies-ruo/apc-cy-7-rat-anti-cd11b.557657
GR1 (RB6-8C5, BioLegend) https://www.biolegend.com/ja-jp/products/alexa-fluor-594-anti-mouse-ly-6g-ly-6c-gr-1-antibody-9672
TCRbeta (H57-597, BioLegend) https://www.biolegend.com/fr-fr/products/pe-cyanine7-anti-mouse-tcr-beta-chain-antibody-4144
CD19 (1D3, BioLegend) https://www.biolegend.com/de-de/products/alexa-fluor-700-anti-mouse-cd19-antibody-22036
TER-119 (TER-119, BD Pharmingen) https://www.bdbiosciences.com/en-au/products/reagents/flow-cytometry-reagents/research-reagents/single-color-antibodies-ruo/biotin-rat-anti-mouse-ter-119-erythroid-cells.553672
CD138 (281-2, BD Pharmingen) https://www.bdbiosciences.com/en-au/products/reagents/flow-cytometry-reagents/research-reagents/single-color-antibodies-ruo/pe-rat-anti-mouse-cd138.561070
CD38 (Ab90, BD Pharmingen) https://www.bdbiosciences.com/en-au/products/reagents/flow-cytometry-reagents/research-reagents/single-color-antibodies-ruo/percp-cy-5-5-rat-anti-mouse-cd38.562770
FAS (Jo2, BD Pharmingen) https://www.bdbiosciences.com/en-au/products/reagents/flow-cytometry-reagents/research-reagents/single-color-antibodies-ruo/pe-cy-7-hamster-anti-mouse-cd95.557653
CD43 (S7, WEHI) Wells SM, Kantor AB, Stall AM. CD43 (S7) expression identifies peripheral B cell subsets. J Immunol. 1994 Dec 15;153(12):5503-15. PMID: 7989752.
Cas9 (7A9-3A3, Cell Signaling Technology) https://www.cellsignal.com/products/antibody-conjugates/cas9-7a9-3a3-mouse-mab-pe-conjugate/35193
BCL-2 (BCL2/10C4, BioLegend) https://www.biolegend.com/it-it/products/pe-anti-bcl-2-antibody-6466
BIM (3C5, WEHI) Hockings C, Alsop AE, Fennell SC, et al. Mcl-1 and Bcl-xL sequestration of Bak confers differential resistance to BH3-only proteins. Cell Death Differ. 2018;25(4):721-734. doi:10.1038/s41418-017-0010-6

Primary antibodies used for Western blotting:
HSP70 (N6, Gift, Dr W. Welch, USCF) Ellis S, Killender M, Anderson RL. Heat-induced alterations in the localization of HSP72 and HSP73 as measured by indirect immunohistochemistry and immunogold electron microscopy. J Histochem Cytochem. 2000;48(3):321-332. doi:10.1177/002215540004800302
beta-Actin (13E5, Cell Signaling Technology) https://www.cellsignal.com/products/primary-antibodies/b-actin-13e5-rabbit-mab/4970
Mouse BCL-2 (3F11, BD Pharmingen) https://www.bdbiosciences.com/en-au/products/reagents/flow-cytometry-reagents/research-reagents/single-color-antibodies-ruo/purified-hamster-anti-mouse-bcl-2.554218
Human/mouse BCL-2 (7/Bcl-2, BD Transduction Laboratories) https://www.bdbiosciences.com/en-au/products/reagents/microscopy-imaging-reagents/immunofluorescence-reagents/purified-mouse-anti-bcl-2.610539
TRP53 (CM5, Novocastra) https://shop.leicabiosystems.com/us/ihc-ish/ihc-primary-antibodies/pid-p53-protein-cm5
BIM (polyclone, Enzo) https://www.enzolifesciences.com/ADI-AAP-330/bim-bod-polyclonal-antibody/
MCL-1 (14C11-20, Gift, DCS Huang, WEHI) Brennan MS, Chang C, Tai L, et al. Humanized Mcl-1 mice enable accurate preclinical evaluation of MCL-1 inhibitors destined for clinical use. Blood. 2018;132(15):1573-1583. doi:10.1182/blood-2018-06-859405
BCL-XL (9C9, WEHI) Grabow S, Kueh AJ, Ke F, et al. Subtle Changes in the Levels of BCL-2 Proteins Cause Severe Craniofacial Abnormalities. Cell Rep. 2018;24(12):3285-3295.e4. doi:10.1016/j.celrep.2018.08.048
A1 (6D6, WEHI) This antibody was validated in this paper (Fig.7c) as A1 expression was increased when cells were transduced with A1 SAM sgRNA.

Eukaryotic cell lines

Policy information about cell lines and Sex and Gender in Research

Cell line source(s)

293T were purchased from ATCC. The Eμ-Myc lymphoma-derived cell lines EMRK-1184 and MRE-721 have been decribed in Baell JB, et al. Nature. 2018 Aug;560(7717):253-257 and Kotschy, A et al. Nature. 2016 Oct 27;538(7626):477-482, respectively. The Eμ-Myc/dCas9a-SAMKI/+/sgBcl-2 (or sgMdm2, or sgNT) lymphoma cell lines were derived from tumour tissues of female mice. Primary B cells or T cells were generated from splenocytes of WT or dCas9a-SAM KI mice (both genders). HSPCs and MEFs were generated from E14.5 embryos of dCas9a-SAMKI/+ mice.

Authentication

Cell lines were authenticated by their morphology and functions according to ATCC instructions or published studies. No additional specific authentication was performed.

Mycoplasma contamination

All cell lines were tested as negative for mycoplasma contamination.

Commonly misidentified lines
(See ICLAC register)

No commonly misidentified line was used.

Animals and other research organisms

Policy information about studies involving animals; ARRIVE guidelines recommended for reporting animal research, and Sex and Gender in Research

Laboratory animals

All mice are Mus musculus maintained on a C57BL/6 background. For haematopoietic cell analysis and tissue westerns, WT, dCas9a-SAMKI/KI mice of 8-week old male mice were used. For haematopoietic reconstitution and MEF generation, 10-20-week old Eμ-Myc male mice and dCas9a-SAMKI/KI female mice were intercrossed to obtain Eμ-Myc;dCas9a-SAMKI/+ embryos . Fresh E14.5 foetal liver cells (a rich source of HSPCs) of Eμ-Myc;dCas9a-SAMKI/+ embryos were used. For haematopoietic reconstitution, 6-week old female recipient mice were used.

Wild animals

The study did not involve wild animals.

Reporting on sex

No sex-based analysis was performed.

Field-collected samples

The study did not involve samples collected from the field.

Ethics oversight

All mouse experiments were performed according to the guidelines of the animal ethics committee of WEHI.

Note that full information on the approval of the study protocol must also be provided in the manuscript.

Flow Cytometry

Plots

Confirm that:

☒ The axis labels state the marker and fluorochrome used (e.g. CD4-FITC).

☒ The axis scales are clearly visible. Include numbers along axes only for bottom left plot of group (a 'group' is an analysis of identical markers).

☒ All plots are contour plots with outliers or pseudocolor plots.

☒ A numerical value for number of cells or percentage (with statistics) is provided.

Methodology

Sample preparation	For the surface staining, cells were washed and resuspended in 50 µL of FACS Buffer (5% FBS, 5 µM EDTA in PBS) with diluted antibodies for 30 minutes on ice in the dark. Cells were washed once in FACS buffer before analysing. Staining with PI (1 µg/mL) was used to exclude dead cells. For the intracellular staining, cells were fixed and permeabilized and then stained using the Intracellular Fixation & Permeabilization Buffer Set according to the manufacturer's instructions.
Instrument	Flow cytometry was performed using BD LSR IIW or LSR Fortessa X20.
Software	Flow cytometry data was acquired using BD FACSDiva software v8.0. Data analysis was preformed using FlowJo v10.
Cell population abundance	Virally transduced cells were sorted using BD Aria W by enriching for eGFP-positive cells.
Gating strategy	The flow cytometry profiles were gated preliminary on FSC-A/SSC-A lymphocytes population, then on FSC-A/FSC-H for single cell population, then on PI- for live cells. Additional gatings were performed as described in figures or figure legends for individual experiments.

☒ Tick this box to confirm that a figure exemplifying the gating strategy is provided in the Supplementary Information.
